# Effects and synergy of feed ingredients on canine neoplastic cell proliferation

**DOI:** 10.1186/s12917-016-0774-9

**Published:** 2016-08-02

**Authors:** Corri B. Levine, Julie Bayle, Vincent Biourge, Joseph J. Wakshlag

**Affiliations:** 1 Department of Clinical Sciences, Cornell University College of Veterinary Medicine, Veterinary Medical Center C2-009, Ithaca, 14853 NY USA; 2 Royal Canin Research Center, Airmargues, France

**Keywords:** Curcumin, Rosemary, Canine cancer, Mastocytoma, Mammary carcinoma, Osteosarcoma, Cell proliferation

## Abstract

**Background:**

Adjunctive use of nutraceuticals in human cancer has shown promise, but little work has been done in canine neoplasia. Previous human research has shown that polyphenols and carotenoids can target multiple pathways in vitro and in vivo. These compounds could synergize or antagonize with currently used chemotherapies, either increasing or decreasing the effectiveness of these drugs. Considering the routine and well controlled feeding practices of most dogs, the use of nutraceuticals incorporated into pet food is attractive, pending proof that the extracts are able to improve remission rates. The aim of this study was to examine five feed ingredients for antiproliferative effects, as well as the interaction with toceranib phosphate and doxorubicin hydrochloride, when treating canine neoplastic cell lines in vitro.

**Results:**

Screening using MTT proliferation assays showed that green tea, turmeric, and rosemary extracts were the most effective. Turmeric extract (TE) was the most potent and exhibited synergy with a rosemary extract (RE) at concentrations from 1 to 25 μg mL^−1^. This combination had an additive or synergistic effect with chemotherapeutic agents at selected concentrations within each cell line. No significant effects on cell viability were observed when the combination therapy was used with normal primary cells.

**Conclusions:**

The use of turmeric and rosemary extracts in combination may be worthwhile to investigate in the pre-clinical and clinical neoplastic considering there are no negative effects on traditional chemotherapy treatment. Further studies into the pharmacokinetics and mechanisms of action of these extracts should be investigated.

## Background

Neoplasia is the cause of death in 20–30 percent of dogs in the United States, and the incidence and mortality increases to 50 % in dogs over the age of ten [1–3]. Risk factors for developing cancer vary based on age, breed, sex, medical history, and nutrition [4]. The incidence and, in some cases, tumor origin are different between dogs and humans, with lymphoma, mast cell disease, osteosarcoma, and mammary neoplasia being particularly prevalent in dogs [5]. Current treatments rely on the use of chemotherapeutics, radiation therapy, and, when practical, surgical interventions. The efficacy of such treatments varies greatly and relies heavily on the stage of cancer progression when treatment is provided, genetic predispositions, and tolerance to the chemotherapy and/or radiation treatments.

Most chemotherapeutic treatments are limited and target DNA replication (cell cycle), cellular metabolism or a single regulatory pathway. In the case of high grade and refractory tumors, multiple drug combinations are often used in order to achieve remission. As the number of treatment modalities increase there can be compounding side effects that are not always well tolerated by patients [6].

The use of natural compounds, or nutraceuticals, in combination with traditional chemotherapy might provide value, and 40 % of pet owners admit to using nutritional supplements as a complementary therapy [7]. Natural products have been used in the initial design of chemotherapies, with well over half of all compounds synthesized or screened being more potent derivations of natural products [8]. The effects of these compounds, whether natural or synthetically made, act through a variety of mechanisms that may contribute to the anti-cancer properties [9, 10]. In fact, a single compound might be able to induce apoptosis through several different pathways [11]. Much of the natural product research performed has been on human or rodent cell lines, particularly of epithelial origin, with little research being done on canine cell lines [12]. More research is necessary to determine the specific effects of these natural products in canine cells and in combination with chemotherapies commonly used in veterinary medicine, as there is a paucity of information in veterinary medicine [13–15].

Plant extracts consist of various polyphenols, terpines, stilbenoids and carotenoids [16]. Several epidemiological studies in humans have found benefits to the inclusion of polyphenol and carotenoid rich fruits and vegetables in the diet [17, 18]. A case–control study has been completed in dogs, and the results indicated an inverse relationship between the consumption of vegetables and the risk of developing transitional cell carcinoma of the urinary bladder in Scottish Terriers [19]. The potential use of certain polyphenols and carotenoids as a treatment option is ongoing in pre-clinical trials with treatment of human epithelial carcinomas on the horizon [20–22]. However; it is necessary to investigate the potential effectiveness in canine cancers which represent other cellular lineages with round, mesenchymal and epithelial neoplasias all being prevalent. In veterinary patients, there have been few intervention studies to examine nutraceutical dietary additions, with two studies examining possible treatment of lymphoma in dogs [23, 24]. Considering that the typical daily feeding patterns of companion dogs are consistent (once or twice per day), the potential for incorporation of natural anti-carcinogenic ingredients is feasible, so long as the added herbal, fruit, or vegetable extract is considered generally reliable and safe by the American Association of Feed Control Officials [25].

The objective of this study was to examine the antiproliferative effects of five commonly used natural food extracts chosen from an initial cytotoxic screening, which could be utilized in commercial dog food, on canine mastocytoma, mammary carcinoma, and osteosarcoma cell lines. After defining efficacy of the leading extracts we set forth to determine if combinations of the most potent extract, turmeric extract [85 % curcuminoids] (TE) could work synergistically with the other extract, and whether these extracts had an additive effect when used in conjunction with the chemotherapeutic drugs most often utilized in the treatment of each neoplastic disease represented in the cell culture systems.

## Methods

### Substances

Natural extracts were received directly from the manufacturer and the content of each compound of interest based on the manufacturers’ purity analysis was verified by a secondary laboratory (Table 1). Extracts were dissolved at 20 mg mL^−1^ in 100 % dimethyl sulfoxide (DMSO; Sigma-Aldrich, St. Louis, MO, USA) to obtain stock solutions before every experiment. Chemotherapeutic agents used were toceranib phosphate (Palladia^TM^, Zoetis Animal Health, Florham Park, NJ) and doxorubicin hydrochloride (Sigma Aldrich, St Louis, MO); fresh dilutions were made from stock before each experiment.

**Table 1:** Characteristics of natural extracts

Common name	Latin name	Part used	Compound of interest	Purity^a^	Manufacturer	Product name	Product number	Batch/Lot number
Black pepper	*Piper nigrum*	Fruit	Piperine	95.02 %	Sabinsa	VetPerine	FP-0215–06	C130329
Green tea	*Camellia sinensis*	Leaf	EGCG	45.76 %	Naturex	Green tea extract	EA140362	A043/008/A13
Pomegranate	*Punica granatum*	Skin	Punicalagins	35.60 %	Polinat	Pomegranate extract [40 % punicosides]	P40P	P40P13–2102
Rosemary	*Rosmarinus officinalis*	Leaf	Carnosic acid	66.90 %	Vitiva	Rosemary extract INOLENS70	302036	LAB.13–036001
Turmeric	*Curcuma longa*	Root	Curcuminoids	87.59 %	Naturex	Turmeric extract	DA251471	A060/006/D13

^a^Purity value represents the percent of the main compound of interest in each extract as determined by manufacturer

#### Cell culture

Three canine neoplastic primary cell lines were used, representing round, mesenchymal and epithelial tumor types: mastocytoma C2 (University of California, San Fransisco, USA), mammary gland carcinoma CMT-12 (Auburn University, Alabama, USA), and osteosarcoma D17 (#CCL-183; ATCC, Manassas, VA, USA). The cell lines were grown on tissue culture-treated plates (Laboratory Product Sales [LPS], Rochester, NY, USA) with appropriate medium containing 10 % heat inactivated fetal bovine serum (HI-FBS; Invitrogen, Carlsbad, CA, USA) and 1 % antibiotic-antimycotic (Invitrogen). Cell lines were grown at 37 °C and 5 % CO_2_ for all experiments and passage of cells, unless otherwise noted. Canine primary dermal fibroblasts (Applied Biological Materials [ABM], Richmond, BC, Canada) were used to investigate effects on normal cells and were propagated and maintained on PriCoat T25 flasks (ABM) in Prigrow II medium (ABM) containing 10 HI-FBS (Invitrogen) and 1 % penicillin/streptomycin (Invitrogen).

#### MTT proliferation assay

Cells were plated at a density of 4 × 10^3^ cells per well on 96-well tissue culture-treated flat bottom plates (LPS) and incubated overnight in complete medium. Cells were treated the following day with DMSO vehicle control or extract using a twofold serial dilution for eight final concentrations ranging from 0.4 to 100 μg mL^−1^ for 48 h representing physiological and supra-physiological concentrations to assess all extracts for potential effectiveness at reducing cellular proliferation. A twofold serial dilution of chemotherapy was also tested ranging from 1.7 to 100 nM toceranib phosphate for the C2 cell line, and 0.03 to 2 μM doxorubicin hydrochloride for the CMT-12 and D17 cell lines. To quantify cellular proliferation, 3-(4,5-dimethylthiazol-2-yl)-2,5-diphenyltetrazolium bromide (MTT dye; Alfa Aesar, Ward Hill, MA, USA) assays were performed by adding 30 μL of MTT dye (5 mg mL^−1^ in phosphate-buffered saline solution) to each well and incubating at 37 °C for 1 h. Media were then aspirated and the cells were solubilized in 200 μL of isopropanol. The optical density of each well was analyzed on a spectrophotometric plate reader (Epoch; Biotek, Winooski, VT, USA) at a wavelength of 570 nm as previously described [26]. Single extract experiments were assayed in duplicate in four independent experiments.

Synergy between extracts was examined using combinations of two extracts at six concentrations: 0.8, 1.7, 3.1, 6.3, 12.5, or 25 μg mL^−1^ as a representation of potentially high end physiological concentrations. All pairwise combinations of extract concentrations were tested. The percent proliferating cells of control for each treatment was pooled from all experiments and is reported as mean ± standard error of the mean.

#### Interaction with toceranib phosphate/doxorubicin hydrochloride using proliferation assay

Cells were plated at a density of 4 × 10^3^ cells per well on 96-well tissue culture-treated plates (LPS) and incubated overnight in complete medium. Cells were treated the following day with 50 μL of a combination of TE (0.8, 1.7, 3.1, or 6.3 μg mL^−1^), RE (0.8, 1.7, 3.1, or 6.3 μg mL^−1^), and chemotherapeutic drug. The C2 cell line was treated with toceranib phosphate (3.1, 6.3, 12.5, 25, 50, 100 nM) and the CMT-12 and D17 cell lines were treated with doxorubicin hydrochloride (0.03, 0.06, 0.13, 0.25, 0.5, 1, 2 μM). DMSO was used as a vehicle control for all treatments. Cells were treated for 48 h before performing MTT assays. Wells treated with DMSO were considered to represent 100 % proliferating cells. All combinations were tested in duplicate in two independent experiments.

#### Trypan blue exclusion assay of cell viability

The trypan blue exclusion assay was performed on canine primary dermal fibroblasts (CDF) due to the slow rate of proliferation and low metabolic activity of these normal canine cells, precluding productive MTT assays. The effects of extract treatments were compared to the results obtained on the C2, CMT-12, and D17 cell lines. For the CDF cells, Applied Cell Extracellular Matrix (ABM) was applied overnight to 24-well tissue culture-treated plates (LPS). For all cell lines, cells were plated at a density of 5 ×10^3^ cells per well and incubated until 60 % confluent before treatment with DMSO vehicle control, 6.3 μg mL^−1^ TE, 6.3 μg mL^−1^ RE, or a combination of 3.1 μg mL^−1^ each of TE and RE. After 48 h of treatment, cells were collected and centrifuged at 1,900 g for 10 min. With the exception of the C2 cell line, cells were detached with 0.05 % Trypsin/EDTA. The cell pellet was resuspended in 0.1 % trypan blue (Sigma) in phosphate-buffered saline solution and 1 % FBS, loaded on a hemocytometer, and visualized on an inverted microscope. Cells which stained blue were considered non-viable. All treatments were performed in triplicate and the percent of viable cells were averaged. All values were standardized to the vehicle control treatment which was considered to represent 100 % viable cells.

#### Data management and calculations

Raw data from proliferation assays (optical density of each well) were normalized to the vehicle alone treatment for individual assays, considered to represent 100 % proliferating cells (single or combined treatment). The percent proliferating cells was then averaged across each replicate. The IC_50_ for each extract was then calculated across experiments by Probit analysis.

The compound interactions were calculated by multiple drug effect analysis using CalcuSyn software (v.2.11; Biosoft, Cambridge, GB, United Kingdom) which employs the median equation principle according to the methodology described by Chou and Talalay [27] to determine a combination index (CI) value by the formula:

$$ CI=\frac{(D)_1}{{\left({D}_x\right)}_1}+\frac{(D)_2}{{\left({D}_x\right)}_2}+\frac{(D)_1{(D)}_2}{{\left({D}_x\right)}_1{\left({D}_x\right)}_2} $$

Where (D)_1_ and (D)_2_ are the doses of both compounds in combination and (D_x_)_1_ and (D_x_)_2_ are the doses of each compound alone at x percent of inhibition. CI values ≤0.9 indicate synergism, a CI value >0.9 and <1.1 indicates an additive effect, and CI values ≥1.1 indicate antagonism.

#### Statistical analysis

All statistical analysis on the outcome of percent proliferating cells as measured by MTT assay was performed using JMP Pro (v. 11.2.1; SAS Institute Inc., Cary, NC, USA). The residuals of the statistical model were evaluated for normality and found to be not normally distributed. Therefore, non-parametric Kruskal-Wallis test was used to compare differences in percent proliferating cells for every treatment concentration used within each cell line across experiments. Comparisons between each treatment group and vehicle control group were carried out using the Steel method adjusting alpha risk for multiple comparisons.

For the outcome of percent viability determined by the trypan blue exclusion assay, residuals of the statistical model were found to be normally distributed, and therefore analyzed using analysis of variance with Tukey’s method for comparison between all treatments, controlling for multiple comparisons. Differences were considered statistically significant at *p* < 0.05.

## Results

### Single treatment versus dual combination treatment on three types of cancer cell lines

All three cell lines were most sensitive to TE, with an IC_50_ below 13 μg mL^−1^ and a significant decrease in cell proliferation at concentrations of at least 0.8 μg mL^−1^ in the C2 cell line, and at concentrations of 6.3 μg mL^−1^ and higher in both the CMT-12 and D17 cell lines (*p* < 0.0001). RE also had an effect with IC_50_ less than 14 μg mL^−1^ in all three cell lines, and a significant decrease in cell proliferation at a concentration of 6.3 μg mL^−1^ and above in the C2 cell line (*p* = 0.0203) and at 12.5 μg mL^−1^ and above in the CMT-12 and D17 cell lines (*p* < 0.0001). Low concentrations of RE caused a minor increase in proliferation when used alone: 9 % increase with 0.8 μg mL^−1^ in C2; an average of 11 % increase with concentrations of 3.1 μg mL^−1^ and below in CMT-12; average of 12 % increase with concentrations of 3.1 μg mL^−1^ and below in D17 (*p* < 0.05). Green tea, black pepper, and pomegranate [40 % punicosides] extracts required a concentration greater than 20 μg mL^−1^ to reduce cell proliferation (Table 2).

**Table 2:** IC_50_ of the extracts and chemotherapy determined by MTT assays (single treatment)

	Green tea extract	Pomegranate extract [40 % punicosides]	Rosemary extract [70 % Carnosic Acid]	Turmeric extract	Black pepper extract	Chemotherapy^a^
	IC_50_ (μg/mL)	IC_50_ (μg/mL)	IC_50_ (μg/mL)	IC_50_ (μg/mL)	IC_50_ (μg/mL)	IC_50_
C2	11.5	48.5	11.9	4.8	21.8	12.5 nM (6.2 ng mL^−1^)
CMT-12	20.4	40.9	13.0	9.1	34.5	0.3 μM (163.1 ng mL^−1^)
D17	47.8	93.8	13.6	12.3	36.5	0.5 μM (271.8 ng mL^−1^)

Values were determined by averaging duplicate wells in four independent experiments and using Probit analysis

^a^C2 treated with toceranib phosphate, CMT-12 and D17 treated with doxorubicin hydrochloride

#### Turmeric extract (TE) and rosemary extract [70 % carnosic acid] (RE) combination

**Fig. 1:**
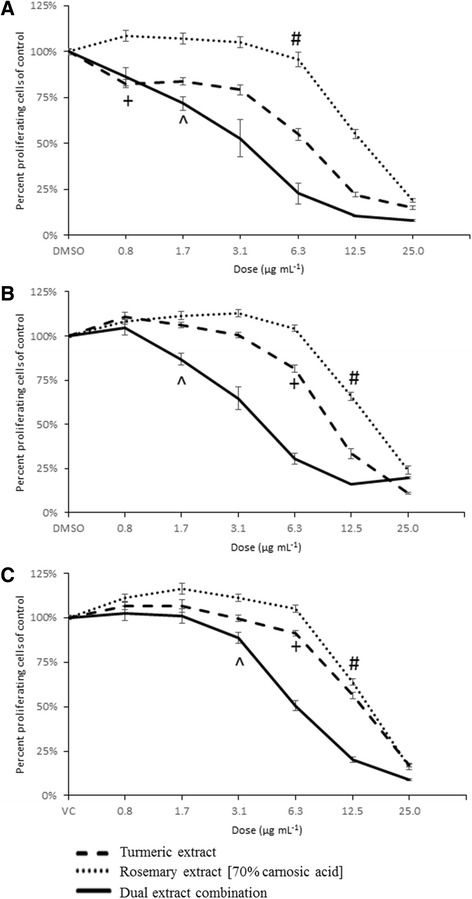
Synergistic combinations of TE and RE. Percent proliferating cells of control are represented as mean ± SEM and were determined by MTT assay on C2 (**a**), CMT-12 (**b**), and D17 (**c**) cell lines. TE and RE were used individually (dashed lines) or in combination at equal concentrations (solid line) at doses ranging from 0.8 to 25 μg mL^−1^. Lowest dose that induced a significant (*p* < 0.05) decrease in percent proliferation compared to DMSO control indicated by + (TE alone), # (RE alone), and ^ (dual extract combination)

The most effective and consistently synergistic combination was between TE and RE. No other dual extract treatments were found to have considerable or consistent synergy at any combination of extract across all three cancer cell lines (data not shown for 4 negative combinations). The combination of TE and RE resulted in a decrease in the concentrations of each extract needed to reach an IC_50_ in all three cell lines suggesting a synergistic combination (Fig. 1a-c). The IC_50_ required in combination (determined using equal concentrations of extracts) was 2.9 μg mL^−1^ of each extract in C2, 4.9 μg mL^−1^ of each extract in CMT-12, and 7.5 μg mL^−1^ of each extract in D17. Analysis using Calcusyn software resulted in Combination Index (CI) values below 0.9, indicating synergy, in treatment concentrations at or below 12.5 μg mL^−1^ of each extract in all three cell lines (Table 3A-C). With treatment using this combination, a significant decrease in cell proliferation compared to vehicle control was first observed at a concentration of 1.7 μg mL^−1^ TE + 1.7 μg mL^−1^ RE in the C2 (IC_28_, *p* = 0.0001) and CMT-12 (IC_13_, *p* = 0.0019) cell lines, and at a concentration of 3.1 μg mL^−1^ TE + 3.1 μg mL^−1^ RE in the D17 cell line (IC_11_, *p* = 0.0073). Once a concentration of 6.3 μg mL^−1^ TE + 6.3 μg mL^−1^ RE was reached in the C2 cell line (*p* < 0.0001) and a concentration of 12.5 μg mL^−1^ TE + 12.5 μg mL^−1^ RE in the CMT12 (*p* = 0.0052) and D17 (*p* = 0.0041) cell lines, further increases in inhibition were not detected with higher concentrations when compared to DMSO treatment alone.

**Table 3:** Combination Index for two extract treatment

Combination Index (CI) values for C2 (A), CMT-12 (B), and D17 (C) cell lines treated with TE and RE in combination at doses of 0.8, 1.7, 3.1, 6.3, 12.5, and 25.0 μg mL−1. CI values ≤ 0.9 indicate synergism (bold values), a CI value >0.9 and <1.1 indicates an additive effect, and CI values ≥1.1 indicate antagonism (italicized values). NP = no calculation possible due to antagonism with single extract alone (RE)

### Natural extracts and chemotherapy interaction on growth inhibition of cancer cell lines

The CI values generated for the C2 cell line with dual extract treatment in the presence of toceranib phosphate at the IC_25_ (Table 4A) and IC_50_ (Table 4B) showed that when both extracts were added at 0.8 or 1.7 μg mL^−1^ there was a mild antagonistic to additive effect, while at 3.1 μg mL^−1^ of both extracts there was a definitive additive effect. When either extract was added at 6.3 μg mL^−1^ there was a definitive synergistic effect with toceranib phosphate regardless of the amount of the other extract. At the IC_75_ (Table 4C) of toceranib phosphate, synergy was only seen when both extracts were used at 6.3 μg mL^−1^, all other combinations produced an additive or mildly antagonistic effect. When the CMT-12 cell line was treated with an IC_25_ of doxorubicin hydrochloride, synergy or an additive effect could be seen when both extracts were used at a concentration of 1.7 μg mL^−1^ or higher and antagonism was seen when either extract was used at a concentration of 0.8 μg mL^−1^ (Table 4D). When doxorubicin hydrochloride was used at the IC_50_ (Table 4E) or IC_75_ (Table 4F), if either extract was used at 3.1 μg mL^−1^ or lower there was modest antagonism. When extracts were combined at concentrations of 3.1 μg mL^−1^ or higher there was an additive or synergistic effect. The D17 cell line showed a modest additive or synergistic effect at any combination of extracts when used with any IC of doxorubicin (Table 4G-I) in general, with the weakest effect at the IC_50_ with possible mild antagonism at 1.7 and 3.1 μg mL^−1^ (Table 4H).

**Table 4:** Combinatorial effects of TE/RE and chemotherapy on tumor cell proliferation

Combination Index (CI) values on C2 (A-C), CMT-12 (D-F), and D17 (G-I) cell lines treated with TE and RE in combination at doses of 0.8, 1.7, 3.1, and 6.3 μg mL^−1^ in the presence of chemotherapeutic agents. Toceranib phosphate used for C2 cell line at (A) IC_25_ dose of 6.3 nM, (B) IC_50_ dose of 12.5 nM, and (C) IC_75_ dose of 12.5 nM; doxorubicin hydrochloride was used for the CMT-12 cell line at (D) IC_25_ dose of 0.1 μM, (E) IC_50_ dose of 0.3 μM, and (F) IC_75_ dose of 1 μM; doxorubicin hydrochloride was used for the D17 cell line at (G) IC_25_ dose of 0.3 μM, (H) IC_50_ dose of 0.5 μM, and (I) IC_75_ dose of 2 μM. CI values ≤ 0.9 indicate synergism (bold values), a CI value >0.9 and <1.1 indicates an additive effect, and CI values ≥1.1 indicate antagonism (italicized value)

### Cytotoxic activity of TE and RE against cancer cell lines without affecting normal cells

**Fig. 2:**
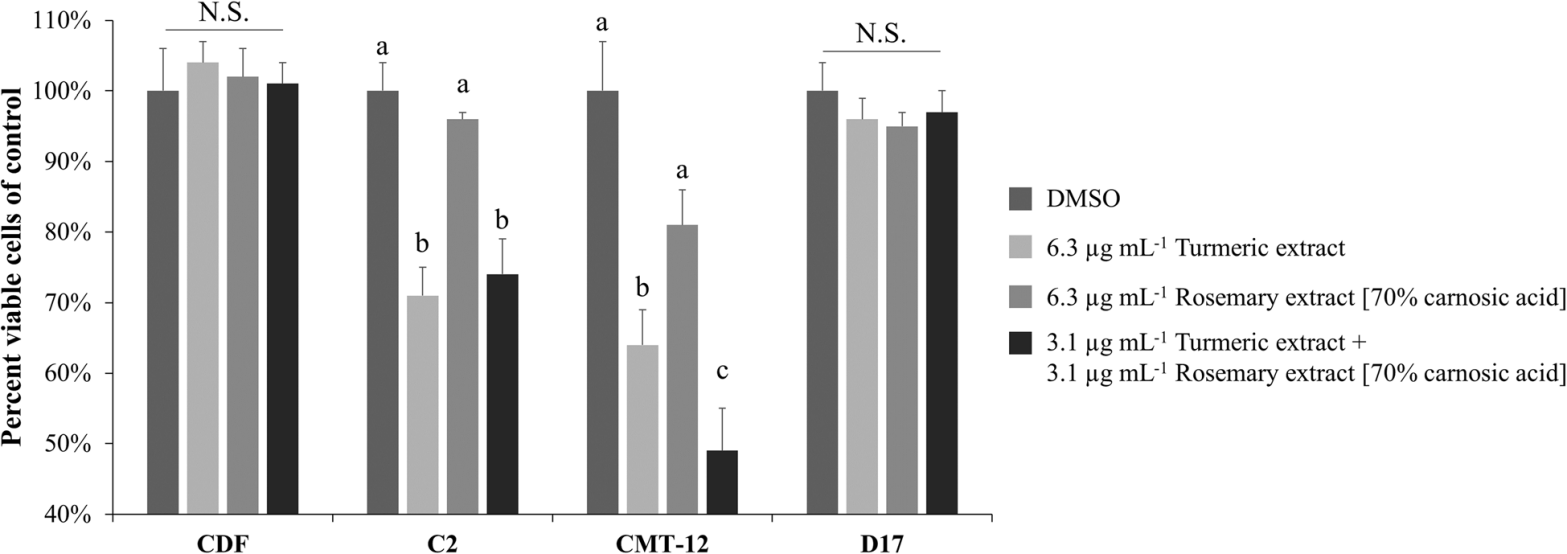
Cytotoxic activity of TE, RE, and combination on tumor cell lines versus normal cells. Percent viable cells determined by trypan blue exclusion assay are represented as mean ± SEM in comparison with DMSO vehicle treatment. Within each cell line, means not sharing the same letter are significantly different (Tukey HSD, *p* < 0.05). NS = Not significant

Figure 2 shows that individual extracts at 6.3 μg mL^−1^ or a combination of 3.1 μg mL^−1^ TE and 3.1 μg mL^−1^ RE did not induce a significant decrease in cell viability in the control primary cells, Canine Dermal Fibroblasts (CDF). In comparison, the three cancer cell lines were also assayed using the same conditions. These concentrations did not induce cytotoxicity on the D17 cell line, while the C2 and CMT-12 cell lines had a significant decrease in cell viability when treated with 6.3 μg mL^−1^ TE alone (29 and 36 %, respectively *p* < 0.01) or with the combination of 3.1 μg mL^−1^ each extract (26 and 51 %, respectively *p* < 0.01).

## Discussion

The use of bioactive polyphenol and/or carotenoids from feed ingredients is well studied in human cancer cells, and there may be efficacy in the utilization of many of these bioactive components, particularly in combination [28, 29]. In the current study we surveyed five extracts which were selected based an initial screen of feed ingredients most abundant anti-proliferative compounds that could hinder cell proliferation at 25 μg mL^−1^, which would represent the high end of physiologically achievable concentration of approximately 10–20 μM for the presumed main component in the extracts examined [14, 15, 30]. Our results indicate that TE was the most potent at inhibiting proliferation at low microgram quantities. To further confirm the single extracts and potential interaction of TE and RE observed in the MTT assay, trypan blue exclusion assays were also performed to assess viable versus non-viable cells. The modest differences in terms of inhibitory effects when comparing the trypan blue and MTT assays may be a reflection of membrane permeability and cytotoxicity being measured with the trypan blue assay versus metabolic cellular activity being measured with the MTT assay. This is particularly evident in the D17 cell line which was less sensitive than the C2 or CMT-12 cell lines, showing diminished activity on MTT proliferative assays and no increase in cell death in the trypan blue assay, suggesting a diminished cell proliferation response rather than cell death response. The response of C2 and CMT-12 cell lines showed that the combination effect of TE with RE was equal, if not superior, in diminishing cell growth as well as increasing the number of non-viable cells than treatment with TE alone, suggesting synergy in this treatment strategy.

Of the two plant extracts that were most effective, TE, which contains 87 % curcuminoids, showed the most potent anti-proliferative effects. These effects could be seen at a concentration below 15 μg mL^−1^ in all cell lines examined. The high potency of curcumin may be related to its binding affinity for at least 30 different proteins [31]. The anti-proliferative effects have been examined in cell culture models of nearly every type of neoplastic condition including leukemia, breast, prostate, bladder, melanocyte, skin, ovarian, hepatic and uterine cancer cells [32]. In most cell-based models, low micromolar concentrations of curcumin thought to be physiological, affect various intracellular pathways ranging from transcriptional activation, to induction of apoptosis, to halting of the cell cycle [33]. These include: transcriptional activators AP-1, Nf-kB, β-catenin, STAT-3, hypoxia induced factor-1, and notch-1; receptor signaling cascades EGF, HER2, VEGF, PDGF, IGF, and FGF; all three major MAP Kinases ERK1/2, p38, and JNK and protein kinase C; intrinsic mitochondrial apoptosis induction by changes in mitochondrial membrane permeability via Bcl-2 and Bax family member proteins are also documented [34]. The wide array of molecular targets has led to over 100 clinical trials in humans to study the use of curcumin to treat various pathologies from obesity related diseases to neurological diseases to various models of neoplastic conditions [35]. Although curcumin and turmeric extracts are effective in vitro, the bioavailability and absorption of curcuminoids is somewhat limited. Typically, less than 10 % of curcumin is absorbed, even with aggressive treatment regimens, leading to high nanomolar serum concentrations in rodents and humans [36, 37]. The curcumin that is absorbed is quickly glycosylated, sulfated, or hydroxylated, and it is unclear if these metabolites are as effective as the unconjugated curcumin [38, 39]. Several approaches to increase bioavailability are being examined including the use of curcumin analogs [40], liposomal curcumin [41], curcumin nanoparticles [42], and adjuvant therapy [54] with bioavailability enhancers such as piperine from black pepper extract [43]. These modifications have led to increased blood curcumin concentrations and a half-life of nearly 10 h. Recently, there have been several studies looking into using whole turmeric extract mixtures instead of pure curcumin alone. Specifically, extract formulations including turmerones has led to increased solubility, absorption, and bioavailability [44]. Generally, treatment with curcumin has exhibited no adverse side effects even at high doses and because of this, a maximum tolerated dose has not been established [45]. Only two canine studies have been completed, with low oral dosing regimens of 4 mg kg^−1^ given orally twice a day showing no side effects after 2 months of treatment [46, 47]. These studies did not measure specifically measure bioavailability, but one study was able to measure transcriptional changes after 20 days of curcumin supplementation [46].

Rosemary extract rich in carnosic acid (RE) also generated interesting results considering the IC_50_ for cellular proliferation is likely within physiological ranges, but more specifically because of its synergistic effects with TE. *Rosmarinus officinalis* contains several phenolic compounds including carnosic acid, carnosol, and rosmarinic acid [48]. In our study, as well as others, carnosic acid and carnosol were more potent in decreasing cellular proliferation than rosmarinic acid in various types of cancer cell lines at concentrations below 20 μM [49, 50]. Carnosic acid and carnosol have been shown to have several mechanisms of action including cell cycle arrest, induction of apoptosis, free radical scavenging, inhibition of metastatic markers, and inhibition of P-glycoprotein mediated drug efflux [51–53]. Intracellular pathways affected include inhibition of PI3-Kinase/AKT/Nf-kB signaling [54], down-regulation of cyclins A and B [55], induction of apoptosis by decreases in Bcl-2 [56], and inhibition of all three major MAP Kinases ERK1/2, p38, and JNK [57]. In rodent studies, the use of a topical [58] or oral [59] rosemary extract has been well tolerated and effective. Toxicity studies in rats have shown that up to 3 g kg^−1^ of rosemary oil is acceptable [60, 61] and biologically relevant levels of around 10 μM can be reached through dietary administration [62], however canine studies are lacking.

We found synergy between TE and RE, which agrees with previous in vitro studies using the same combination [63, 64]. While RE alone was only effective at concentrations above 6.3 μg mL^−1^ in all three cancer cell lines, its use with TE significantly decreased the concentrations needed to reduce cell proliferation. In all three tumor cell lines, these extracts worked synergistically at concentrations between 1 – 10 μg mL^−1^ of each extract. When used in combination, extrapolation of our data accounting for the percentage of the compound of interest (curcumin and carnosic acid) suggest that the IC_50_ is 6.8 μM curcumin and 7.6 μM carnosic acid for C2, 12 μM curcumin and 13 μM carnosic acid for CMT-12, and 18 μM curcumin and 20 μM carnosic acid for D17. Neither of the extracts, when used alone or in combination, showed effects on cell viability in the normal canine dermal fibroblasts, suggesting the effects on normal cell death or proliferation is minimal. Other control cells were considered, including the canine fibroblast A-72 tumor cell line and Madin-Darby Canine Kidney (MDCK) epithelial cells, but due to the highly proliferative and potentially tumorigenic nature of these cell lines they were not used. CDF cells were chosen due to their seemingly normal phenotype, ease of maintenance, and commercial availability. Further studies could examine the effects on primary lymphocytes or epithelial cells, but these cell types were not available at the time this study was completed.

When the C2 cell line was incubated with the TE/RE combination in the presence of toceranib phosphate, a synergistic or additive effect was seen when either extract was used at 6.3 μg mL^−1^, or when TE was used at 3.1 μg mL^−1^ or higher. When the CMT-12 cell line was treated with the TE/RE combination in the presence of doxorubicin hydrochloride, there was a modest antagonistic effect at lower concentrations of both extracts when used alone (below 3.1 μg mL^−1^ of each), but a synergistic or additive effect could be seen with a higher concentration of 6.3 μg mL^−1^ of both extracts individually. The D17 cell line showed considerable additive and synergistic effects with all extract combinations at the IC_25_ and IC_75_ of doxorubicin hydrochloride in general. Mild antagonism was seen when extracts were used at 3.1 μg mL^−1^ or lower, but this was diminished or absent when either extract reached a concentration of 6.3 μg mL^−1^. Considering these findings, further testing of TE and RE with other chemotherapies to ensure similar synergy, additive, or antagonistic effects is warranted. Furthermore, considering the lack of basic pharmacokinetics with oral TE and RE canine studies are needed to examine whether there feed ingredients would have any utility. The synergistic effect of these compounds with chemotherapies is necessary, not only due to the potential to decrease the administered dose for treatment, but also to examine alterations in chemotherapy metabolism.

Other extracts examined in the MTT assay were VetPerine® (piperine), pomegranate extracts and green tea extracts. Effective IC_50_ for these extracts across the cell lines were typically above 25 μg mL^−1^ which would be considered supraphysiological. This takes into account that most animals cannot reach concentrations greater than approximately 10–20 μM of any specific bioactive component from these extracts when used at relatively large doses for any significant period of time. That said, these compounds were also tested for synergy, antagonism, or additive effects and were not observed to increase TE or RE effectiveness (data not shown) and were discounted for further examination with commonly used chemotherapeutics.

## Conclusions

This study of commonly used feed ingredients showed that a combination of TE and RE diminished the growth of cancer cells. This synergistic effect was observed at 10 μg mL^−1^ and below indicating a potential for physiological effects, however in vivo pharmacokinetic and efficacy studies are needed. Although we are unsure of the bioactive molecules inducing these effects, the high concentrations of curcuminoids and carnosic acid are likely involved. The anti-proliferative effects of chosen chemotherapeutics were not hindered when these extracts were used in combination at concentrations of 3.1 and 6.3 μg mL^−1^ across a range of chemotherapeutic concentrations. Further testing of other chemotherapies with these specific extracts is warranted to ensure no distinct antagonism is evident. In addition, further examination of the potential apoptotic effects and cellular pathways affected by these extracts individually and in combination may be fruitful in determining the similarities and differences of their effects between cell lines.

## Abbreviations

CDF, canine dermal fibroblasts; CI, combination index; DMSO, dimethyl sulfoxide; IC_50_, 50 % inhibitory concentration; MTT, 3-(4,5-dimethylthiazol-2-yl)-2,5-diphenyltetrazolium bromide; RE, rosemary extract [70 % carnosic acid]; TE, Turmeric extract
